# WIC Participants’ Perceptions of the Cash-Value Benefit Increase during the COVID-19 Pandemic

**DOI:** 10.3390/nu14173509

**Published:** 2022-08-26

**Authors:** McKenna M. Halverson, Allison Karpyn

**Affiliations:** Department of Human Development and Family Sciences, Center for Research in Education and Social Policy, University of Delaware, Newark, DE 19713, USA

**Keywords:** WIC, cash-value benefit, fruits and vegetables, purchasing, consumption, diet, variety, healthy

## Abstract

Recent changes to the Special Supplemental Nutrition Program for Women, Infants, and Children (WIC) Cash-Value Benefit (CVB), which provides participants with money to spend on fruits and vegetables, have the potential to reduce disparities in healthy food access and food insecurity that were exacerbated by the COVID-19 pandemic. However, few studies have examined how the changes to the CVB allotment that occurred during the pandemic influenced WIC participants’ perceptions of the benefit or their fruit and vegetable purchasing and consumption. To address this gap, we conducted semi-structured interviews with 51 WIC participants in Wilmington, Delaware. Survey measures included demographic questions, the Hunger Vital Sign food insecurity screener, and open-ended questions regarding perceptions of the CVB increase and its influence on participants’ fruit and vegetable purchasing and consumption. Data were analyzed using a hybrid inductive and deductive coding approach. The results demonstrate that higher CVB allotments increased WIC participants’ purchasing and consumption of fruits and vegetables, increased the frequency of their shopping occasions, and enhanced their dietary variety. Our findings also suggest that WIC participants highly value the increased CVB. Consequently, maintaining the increased CVB allotment could improve the nutritional outcomes of low-income mothers, infants, and children participating in WIC.

## 1. Introduction

In 2022, approximately 1 in 9 individuals, including 1 in 6 children, in Delaware experienced food insecurity [1]. Food insecurity is positively related to a host of negative health outcomes including poor diet quality and unhealthy eating behaviors [2], psychological distress [3], and chronic disease (e.g., diabetes, obesity, and cardiovascular disease) [4]. The Special Supplemental Nutrition Program for Women, Infants, and Children (WIC), which is one of the largest nutrition assistance programs in the U.S., aims to reduce food insecurity and improve healthy food access among low-income pregnant and postpartum women as well as infants and children up to age five by supplementing participants’ dietary intake with healthy foods tailored to their specific needs and offering breastfeeding support, nutrition education, and healthcare referrals [5].

In 2021, WIC served approximately 6.2 million women and children across the U.S. [6]. In Delaware, the program served approximately 19,766 participants in 2022, 15,397 of which were infants and children [5]. By reducing barriers to healthy food access that are common among low-income populations, WIC improves mothers’, infants’, and children’s outcomes in a variety of domains including increasing healthy food intake [7] and lowering rates of obesity [8], as well as higher birthweights [9], improved cognitive development and academic achievement outcomes [10], and fewer behavior problems among children when they enter school [11]. These results are promising as infancy and early childhood are times of significant growth and development which set the stage for future developmental outcomes [12].

In Delaware, approximately USD 2.9 million in additional fruit and vegetable benefits were issued to WIC participants between 1 April 2021, and 31 March 2022 [5]. Cash-Value Benefit (CVB) allotments, which provide participants with money to spend on fruits and vegetables, increased from pre-pandemic levels of USD 9/month for children and USD 11/month for adults, to USD 35/month for both women and children during the summer of 2021, before being adjusted in October to USD 24/month for children, USD 43/month for pregnant and postpartum women, and USD 47/month for breastfeeding women in December 2021 [13]. The increased CVB allotment may help to offset some of the barriers to healthy food access commonly faced by low-income populations such as cost and availability [14,15]. Additionally, the CVB increase has the potential to reduce food insecurity among households with young children which was exacerbated during the COVID-19 pandemic [16].

Research regarding the WIC CVB is sparse. Of the studies that have examined factors related to CVB usage, several evaluated pre-pandemic benefit levels and assessed the benefits’ impact on participants’ perceptions of the in-store benefit redemption process as well as participants’ consumption of fruits and vegetables [17,18,19]. Results from these studies demonstrate that WIC participants generally believed that pre-pandemic CVB levels were insufficient and that the monthly benefit allotment should be increased [17,18]. These studies also demonstrated that participants faced several barriers to CVB redemption including negative interactions with store cashiers, perceived stigma, and confusion regarding stores’ inconsistent redemption rules [17]. Additionally, findings demonstrate that WIC participants who redeem their CVB more frequently are more likely to report consuming more fruits and vegetables than their counterparts who redeem the CVB less frequently [19]. Not all studies yielded positive results, however. A study which evaluated whether providing pregnant WIC participants with an additional USD 40 per month via a separate fruit and vegetable voucher, in addition to their pre-pandemic CVB allotment, found no influence of the voucher on participants’ food insecurity or fruit and vegetable intake [20]. However, redemption rates for the voucher program during this study period were lower than pre-pandemic redemption rates (67% vs. 74–81%, respectively), which may have contributed to these null findings.

Few studies to date have evaluated WIC participants’ experiences with CVB redemption after the allotment changes during the COVID-19 pandemic, as well as how the higher allotment influenced fruit and vegetable intake. Findings regarding the influence of the increased CVB allotment on participants’ fruit and vegetable intake, are mixed. Specifically, one evaluation leveraged survey data from four states (i.e., Connecticut, Nevada, New Hampshire, New Mexico) and one Indian Tribal Organization (i.e., Tribal Council of Arizona) to examine WIC participants’ experiences with, and perceptions of, the CVB allotment changes during the COVID-19 pandemic, and found that the higher CVB allotment was significantly associated with a 1/3 cup increase in participants’ daily fruit and vegetable consumption (i.e., from 2.01 cups to 2.31 cups) [21]. In contrast, results from surveys conducted with California WIC participants at two time points (e.g., May 2021 and September 2021) showed that child fruit and vegetable intake significantly decreased following the CVB allotment increase [22].

Regarding WIC participants’ perceptions of the CVB and benefit usage, results from focus groups with WIC participants in North Carolina suggest that WIC participants were not satisfied with pre-pandemic CVB allotments and that they appreciated the allotment increase that occurred during the COVID-19 pandemic [23]. Additionally, survey results from the aforementioned multi-state evaluation showed that most participants (approximately 67%) were pleased with the increased allotment and stated that it was “just right”, whereas 25% said it was not sufficient [21]. Similarly, results from California WIC participants showed that the percentage of participants reporting that the CVB was “not enough” decreased from 89% to 23% following the CVB allotment changes during the COVID-19 pandemic, whereas the proportion of those reporting that the CVB was “just right” significantly increased from 7% to 73% [22]. Participants also expressed that compared with other aspects of the WIC package, they value the CVB because it allows them to get a larger variety of items [23]. Although participants positively perceived the CVB, findings suggested that rural WIC participants had a particularly difficult time redeeming the CVB due to store fruit and vegetable stocking issues and an inability to properly identify WIC-eligible items [23].

Given the limited research regarding the WIC CVB and the mixed evidence regarding the impact of the COVID-19 allotment changes on participant outcomes, this study seeks to characterize how changes to the CVB allotment during the pandemic impacted Delaware WIC participants’ perceptions of the benefit as well as their grocery purchases, food preparation, and fruit and vegetable consumption patterns.

## 2. Materials and Methods

### 2.1. Participants and Procedure

Participants were 53 WIC-eligible individuals recruited from a large regional supermarket chain, child care centers, churches, and community-based organizations in Wilmington, Delaware between March and June of 2022. To be eligible for the study, participants had to be at least 18 years of age, a Delaware resident, English-speaking, contribute to household food purchasing, and a WIC participant between March 2020 and June 2022. Two participants were excluded from analyses; one did not participate in WIC during the study period, and one did not receive the CVB. Therefore, the total study sample includes 51 WIC-eligible participants residing in Wilmington, Delaware.

The qualitative design incorporated 20- to 30-min, semi-structured phone interviews with WIC participants about their experiences with the CVB during the pandemic. In collaboration with community partners at Conscious Connections, Inc. (Wilmington, NC, USA) and Village Tree, Inc. (Wilmington, NC, USA), non-profit organizations aiming to mitigate food insecurity in the Wilmington community, the research team developed a semi-structured interview guide (a table of topics and example questions is provided in Table 1) to evaluate WIC participants’ perceptions of the WIC CVB changes and how they influenced participants’ fruit and vegetable purchasing and consumption. The principal investigator (MMH) conducted all interviews. Our research partners played an integral role in study recruitment, which helped to form connections with participants. Additionally, in line with recommendations for building rapport with participants in qualitative research, the PI displayed empathy and mutual attentiveness, addressed any reservations reported, accommodated participants’ schedules, “engaged in common grounding behavior” by identifying common interests, and sought out feedback during and after the interviews [24,25]. Participants received a USD 20 ShopRite gift card as compensation for their participation in the survey. All study procedures were reviewed and approved by the Institutional Review Board at the University of Delaware.

### 2.2. Measures

#### 2.2.1. Demographics

Participants completed the following demographic questions: gender, race, ethnicity, educational level, income level, employment status, relationship status, parent age, number of children currently participating in WIC, pregnancy status, number of people living in the household, participants’ WIC participation status, current CVB amount, SNAP participation over the last year, and current SNAP participation.

#### 2.2.2. Hunger Vital Sign

The Hunger Vital Sign [26], a two-item food insecurity (FI) screener, was used to identify families at risk of food insecurity: (1) “Within the past 12 months we worried whether our food would run out before we got money to buy more”, and (2) “Within the past 12 months the food we bought just didn’t last and we didn’t have money to get more”. Response options for both questions were “often true”, “sometimes true”, and “never true”. Responses of “often true” and “sometimes true” for either or both questions were coded as food insecure whereas responses of “never true” were coded as food secure. Compared with the longer 18-item US Household Food Security Scale used by the Current Population Survey, the two-item FI screener has high sensitivity (97%), specificity (83%), and convergent validity, making it an effective substitute tool to annually monitor food-insecurity status [26].

#### 2.2.3. Impact and Perceptions of the CVB Increase

A series of open-ended questions which aimed to evaluate WIC participants’ perceptions of the CVB increase and any resulting changes in fruit and vegetable purchasing and consumption were included as part of the semi-structured interview guide (see Table 1).

#### 2.2.4. Data Analysis

Interviews were audio-recorded, uploaded to Rev.com for transcription, and qualitative data were analyzed using Dedoose (9.0.46 OSX). We employed a hybrid inductive and deductive thematic analysis approach [27]. Using deductive coding strategies, the primary investigator (MMH) developed a codebook based on the semi-structured interview guide to understand WIC participants’ perceptions of specific topics related to the CVB allotment changes during the COVID-19 pandemic (e.g., changes in fruit and vegetable purchasing and consumption). The primary investigator also employed inductive coding strategies to identify emergent themes in the data. The study coauthor (AK) provided input in coding discrepancies and helped to resolve conflicts. Participant demographic characteristics were analyzed using SPSS version 27, IBM, Chicago, IL, USA.

#### 2.2.5. Researcher Positionality

As White, middle-class women who obtained our degrees from predominately White academic institutions, we acknowledge that we will never fully understand the experiences of historically marginalized racial and ethnic groups or families experiencing poverty nor the compounding effects of implicit and explicit racism. However, we are committed to continuously challenging our biases, engaging deeply with these populations, forming reciprocal research partnerships, and ensuring that our findings will directly inform more equitable policies and practices.

## 3. Results

Participants’ socio-demographic characteristics are listed in Table 2. Participants were predominately female (100%), Black (70.6%), employed full- or part-time (56.8%), had incomes under USD 30,000 (68.6%), food insecure (76.5%), with an average of 2.3 children (*SD* = 0.89) participating in WIC. Eleven participants did not experience a change in WIC benefit amounts during the study period because they were enrolled for a time period when benefit amounts were static, and questions regarding CVB shifts were excluded in these cases. Most participants reported that their ideal CVB was greater than their current amount (70.5%), even after the allotment increase during the COVID-19 pandemic. For example, Respondent 7 stated,

“I know maybe $50, like a, even a good even number would help like $50 instead of like the weird $43. Because like I said, fruits do go up in price and it can be a little expensive. So, it’s like if the market change for as far as food cost goes, at least I can afford something for like a week, a week to two weeks compared to this like a week and maybe a week and a half of fruits.”

Qualitative data analysis examining WIC participant perceptions of the CVB allotment increase and its impact on fruit and vegetable purchasing and consumption yielded four overarching themes including: (1) increased purchasing of fruits and vegetables and more frequent shopping occasions, (2) increased consumption of fruits and vegetables, (3) enhanced dietary variety, (4) high participant valuation of the increased CVB allotment (see Table 3 for quotes exemplifying each theme).

### 3.1. Theme 1: Increased Purchasing of Fruits and Vegetables and More Frequent Shopping Occasions

Many participants reported that the higher CVB allotment increased their purchasing power to spend on fruits and vegetables. For example, Respondent 14 said,

“My kids they really like fruits and things like that. So, the lesser amount they couldn’t really get, you know, as much as they get now. Now they enjoy it. […] Not putting like a limit on it. Like you already had two, you know, type of thing.”

Participants also stated that the higher CVB allotment allowed them to increase the frequency of their shopping occasions over the course of the month. For example, Respondent 5 described,

“You’ll be able to use it like multiple times through the month. Cause you don’t go grocery shopping, not everybody goes shopping once per month. So, like you go every two weeks, at least you have more money to get more things. So, you don’t have to be like only eating two strawberries […], you can incorporate more, like you say, only two today in the smoothie. Only one today or two.”

### 3.2. Theme 2: Increased Consumption of Fruits and Vegetables

The increased CVB allotment allowed many WIC participants to eat healthier meals by increasing their fruit and vegetable consumption. For example, Respondent 11 stated:

“When we had a lower amount, we stuck to like salads. But as like I do notice now today that as we were able to have more. I basically make sure that we have a full vegetable at every meal, and I know that’s like a really big deal and it kind of makes me weary that we weren’t having a full fruit and vegetable at every meal. So, now we’ll have a side of like cucumbers with ranch or carrots with ranch, always with our regular meat and drink. We’ll also have like fruit, always for breakfast. Now that’s something that we definitely do now more than we’ve ever done before. And that he has snacks also.”

Participants also stated that they ate less junk food and meat and cheese-based meals following the CVB allotment increase. Respondent 53 said, “*I was able to provide him with like grapes or chopped up apples for snack. Cuz you always have snack on the way home from school. Rather than chips or a cake or something that’s pre-packaged.*” Additionally, Respondent 11 said,

“Yeah, I would add that we definitely ate a lot more of like meat and cheese-based meals and now our meals are so much more diverse. Just because the fact that we have them there encourages all, encourages us all, including my child to be like, okay, I’m gonna try vegetable because you know, it’s on my plate and it’s more of a larger part of my meal than just like one or two. Cause like that was another thing too. There wasn’t enough to share so we kind of like were picking at like a little bit versus now we have plenty of the vegetable or fruit to eat.”

### 3.3. Theme 3: Enhanced Dietary Variety

Participants also described how the higher CVB allotment allowed them to enhance their dietary variety by trying several different types of fruits and vegetables. For example, when asked if a CVB allotment of USD 47, compared with USD 9, would change the types of foods she would buy, Respondent 15 stated,

“I think yeah, $47. It would, it would allow for, I’m not gonna just speak for myself, because this is my profession as well, childcare. It would allow the children to expand their ability or their knowledge base about fruits and vegetables. Because let’s just be honest. If they, if you don’t, if you don’t know what a blueberry tastes like, are you willing to spend like your last $3 to get a blueberry, right. Or if you had WIC, you would say, oh, let me get this on my WIC. If they don’t like it don’t harm, no foul. Same with asparagus or any other type, like pears or apples or any other type of fresh fruit and vegetables, you’re willing to try more in different things.”

Similarly, Respondent 1 expressed,

“Yeah, it was easier. Like I said, it was, it, you, you were able to shop with more, a little more freedom. You had more variety, you know, you didn’t feel like you were limited with just two types of fruits or just two types of vegetables. You were able to actually get like blueberry, strawberries and maybe some zucchini. And there goes dinner or there goes some breakfast. Or there goes with the WIC, you’re able to get yogurt and you can get the berries to go with and then dinner, you have, you know, your broccoli that you could have the fresh fruits and broccoli with, you know, the cheese. They helped make the meal. Yeah. Especially with the fresher fruits and vegetables versus the can.”

In addition to trying new types of fruits and vegetables, the increased CVB allotment allowed WIC participants to buy fruit and vegetables that better aligned with the preferences of individual family members. When asked how the shifts in the CVB impacted her family, Respondent 18 responded,

“Like I said, either, something that might be a little bit more expensive that we normally don’t eat or like something that the kids eat, I don’t eat, then I might go ahead and grab it. Cause I normally try to get stuff that we all can eat. So, if like they want something that I don’t, like cucumbers or something like that, I don’t like cucumbers, so like we might get cucumbers or something else like that, that they can just have on their own.”

Additionally, participants expressed that there was a ripple effect of the increased CVB allotment on the entire family, such that more produce for the child also encouraged others to eat healthier. When asked whether the shifts in the CVB impacted her family,

“So, with that addition, I definitely say, okay, if I start with actually, that’s what I do now, I start with broccoli and then I’m like, okay, then pick a meat and then pick a green. And then every time I do that, I’m able to pick a whole meal. So even collard greens, we do a lot more now. Green beans. Then I go from there and I’m able to plan a more diverse, healthy meal for my family.”

Similarly, Respondent 16 stated, “*Yeah, it does. Because everybody eats our portions and you know, where if it’s a lot or enough, we just, we just share. We just make sure we, all, everybody gets a taste of what’s bought and what’s been made*”.

### 3.4. Theme 4: High Participant Valuation of the Increased CVB Alottment

Participants highly valued the increased CVB allotment and reported that the higher CVB allotment helped themselves and their family in a variety of ways. Several participants appreciated that the higher CVB allotment allowed them to save their own money as well as their Supplemental Nutrition Assistance Program (SNAP) benefits. For example, Respondent 52 stated,

“Well, we try to always eat, actually, she prefers to fruit and the vegetables over junk food. So, we always pretty much buy fruit and vegetables, so for it to not come outta, like we, we exceed the $24, definitely every month. So, I mean, it, it helps that it doesn’t have to come outta my pocket or outta my food stamps because then I can use that for other meals or my meats or something instead.”

Many participants also reported that the CVB was their favorite part of the WIC program. As Respondent 25 put it,

“Yeah, I mean, this will only be, I think my second or third month using it and be like the produce, like I was saying, my produce balance is, my most sought after, my most desired piece of my WIC, like, Ooh, I got $43 again. I’m gonna go get me some produce cause produce ain’t cheap girl.”

This positive perception of the CVB was also shared by Respondent 32 who expressed,

“At the start of the pandemic, I would say it helped a lot because I was pregnant so it helped me buy things that I would need because I was going to be breastfeeding. So, the fruits and like vegetables helped me throughout my pregnancies.”

Additionally, Respondent 7 said, “*The fruits and vegetables helped the most.*” Respondent 11 also stated that the increased CVB allotment allowed her to enhance her knowledge of the benefits of fruits and vegetables. Specifically, she said,

“Yeah, I think it really did help me to kind of start educating myself on what types of fruits and vegetables we could eat. How it helps our health and like really helps actually.”

Most participants positively viewed the CVB increase and stated that although they may not redeem their full WIC package every month, the CVB was one component of WIC that they always redeemed. For example, Respondent 25 said, “Sometimes I don’t get the milk or sometimes I don’t get the beans or something like that, but I always get the produce always. That’s the best part.” Similarly, Respondent 7 stated, “I use it all. That’s the one thing I use up all the time.” However, a few participants stated that, although rare, there were certain circumstances when they were unable to redeem their full CVB. When discussing reasons for CVB under-redemption, Respondent 53 stated, “No, I use it, unless there’s like 50 cents left on it or something still like that but I, I try to use all of it.” Additionally, Respondent 16 expressed, “Only if it was last minute, […] getting my shopping done. It’s been kind of hard. I just recently got some support with getting the kids in the daycare so I can make it just sometimes just the drag, the kids and just trying to just be careful with that COVID and bacteria in the way they do it.”

## 4. Discussion

In this study, we found that WIC participants strongly support the CVB allotment increase that occurred during the COVID-19 pandemic, and that the higher benefit amount positively affected participants’ dietary quality. Specifically, participants reported that the higher CVB allotment allowed them to eat healthier by increasing their purchasing and consumption of fruits and vegetables. Additionally, participants reported consuming a greater variety of fruits and vegetables, which allowed them to better understand their children’s preferences. These findings are important because they could potentially inform policymakers’ future decision as to whether to extend the increased CVB allotment or to revert to pre-pandemic levels.

In addition to our main findings, we also found that benefits of the CVB increase extend beyond the WIC recipient to influence the entire family. For example, participants reported that the higher CVB allotment often promoted healthier eating practices among themselves, their children who participate in WIC, and their children who are not receiving WIC benefits. We also found that the higher allotment allowed WIC participants to increase the frequency of their fruit and vegetable shopping occasions over the course of the month. Many participants stated that this was beneficial, as it allowed them to consume fruits and vegetables more consistently. Additionally, many participants stated that the CVB was their favorite part of the WIC program and that they made a concerted effort to redeem the benefit each month. WIC participants in our study appreciated the higher CVB allotment because it allowed them to save their own money as well as their SNAP benefits. They reported that they could then use that money to buy other essentials such as meats and other non-WIC foods. Another reported benefit of the higher allotment was participants’ ability to individualize their fruit and vegetable purchases to align with their children’s preferences.

Further, most participants reported that they always used, or attempted to use, their CVB. Reasons that WIC participants may not use the full CVB each month included busy schedules, attempting to calculate the benefit amounts while shopping with children, not having a large enough balance for another item (e.g., USD 0.50 left on balance), inability to redeem their CVB at certain stores, misplacing their card, missing the benefit renewal date, or forgetting to redeem part of the benefit.

Previous research regarding the impact of WIC CVB allotment changes during the COVID-19 pandemic on participants’ fruit and vegetable consumption is mixed. Specifically, a study involving surveys at two time points before and after the increase found that the CVB allotment changes were associated with decreases in participants’ fruit and vegetable intake [22], whereas two studies involving focus groups and surveys found that participants’ self-reported fruit and vegetable intake increased after the allotment increase [21,23]. Our findings corroborate the latter studies, demonstrating that participants reported consuming more fruits and vegetables after the CVB allotment increase. Our findings align with prior research suggesting that higher CVB allotments increase WIC participants’ purchasing of fruits and vegetables as well as their dietary variety [23]. Previous studies also demonstrate that the CVB is participants’ most highly valued aspect of the WIC program [23,28], which aligns with the findings in this study. Additionally, prior studies demonstrate that although WIC participants highly valued the CVB, they weren’t satisfied with pre-pandemic CVB allotments [17,18,23]. Our results support this finding and suggest that participants’ desire for a higher CVB remained even after the increases during the COVID-19 pandemic, which supports previous work [23]. This study also expands upon previous research by highlighting the experiences of WIC participants living in an urban city and their ability to increase the frequency of their shopping occasions over the course of the month as well as the benefits of the allotment change for the entire family.

This study has some limitations which are important to highlight. First, our sample included predominately Black mothers living in an urban city in Delaware. As such, results may not be consistent with WIC participants of other races and ethnicities in other geographic regions. Second, we obtained retrospective data from WIC participants on the effect of the increased CVB allotment on their fruit and vegetable purchasing and consumption, which required participants to recall their experiences one to two years prior to the study.

Future studies evaluating fruit and vegetable purchasing occasions and specific groceries purchased with the increased CVB allotment would be beneficial to expand upon the results reported herein. In addition, expanded research should include mixed methods designs and quantitative data related to CVB purchasing frequency, quantities of fruits and vegetables purchased, and fruit and vegetable cost variations, both locally and nationally. Rigorous studies including randomized designs, or which involve natural experiments should be considered. If changes in CVB allocations were to occur differentially by states, this would provide a unique opportunity to evaluate variations in benefit allotment at a much larger scale. More studies are needed to identify additional approaches to enhance the CVB and to understand if strategies like incentives impact CVB-related fruit and vegetable purchasing and diet. Additionally, our study sample was predominately Black, so future studies focusing on other racial and ethnic populations and subpopulations, such as Asian Americans, would be valuable.

## 5. Conclusions

The findings from this study demonstrate that WIC participants strongly support the CVB allotment increase that occurred during the COVID-19 pandemic, and that higher CVB allotments increased participants’ purchasing and consumption of fruit and vegetables as well as their dietary variety. Additionally, our findings suggest that although participants appreciated the higher CVB allotment during the COVID-19 pandemic, many believe that in order to meet their families’ needs, it should be increased further. By advancing policymakers understanding of the beneficial effects of the increased CVB allotment, this study may promote health equity by increasing low-income children and families’ access to nutritious, affordable food.

## Figures and Tables

**Table 1:** Representative Interview Questions.

(1) Can you describe how the shifts in your Cash-Value Benefit amount impacted your family? (2) What do you usually buy with your Cash-Value Benefit? (3) How do you use the items that you purchase with your Cash-Value Benefit? (4) When you have more money to spend as part of the Cash-Value Benefit, how do your purchases change if at all? (5) How does having more money in a Cash-Value Benefit change the way your family eats? For example, do meals or snacks look different? (6) How much money makes a difference? Does a difference between $11/person vs. $43 or $47/person change the kinds of food you buy? If so, how? (7) What would your ideal Cash-Value Benefit be?

**Table 2:** Sample Characteristics (*N* = 51).

	Mean (SD)
Parent Age	30.88 (7.01)
Number of People in Household	4.39 (2.01)
Number of Children Currently Participating in WIC	2.3 (0.89)
Participants’ Current Cash-Value Benefit (CVB) Allotment	35.72 (18.89)
Participants’ Ideal CVB Allotment	45.22 (21.70)
	**%**
Gender (Female)	100
Race	
Black or African American	70.6
White	11.8
Other	2.0
Ethnicity	
Hispanic/Latino	21.7
Educational Level	
Less than a High School Degree	9.8
High School Degree	49.0
Some College	31.4
Bachelor’s Degree	7.8
Doctorate/Professional Degree	2.0
Income Level	
Under $30,000	68.6
$30,000–$60,000	21.6
$60,001–$90,000	3.9
Over $90,000	2.0
Prefer Not to Say	3.9
Employment Status	
Employed Full Time	39.2
Employed Part Time	17.6
Unemployed and Looking for Work	17.6
Unemployed and Not Looking for Work	9.8
Unable to Work/On Disability	9.8
Other	5.9
Relationship Status	
Single	66.7
Live at Home with Partner or Spouse	29.4
Other	3.9
Food Insecure	76.5
Mother Currently Participates in WIC (*N* = 44) *	29.5
Mother is Currently Pregnant	19.6
Currently Enrolled in SNAP (*N* = 42) *	56.9
Enrolled in SNAP Over the Last Year (*N* = 43) *	70.6
Participants Who Would Like Higher CVB Than Current Amount (*N* = 44) **	70.45

* Question added during survey administration. ** Participants who no longer received the CVB or who did not know their current amount were excluded from analysis.

**Table 3:** Qualitative Themes and Exemplar Quotes.

**Theme #1: Increased Purchasing of Fruits and Vegetables and More Frequent Shopping Occasions**	
Increased Purchasing of Fruits and Vegetables	“So, yeah, like before the pandemic, the fruit and vegetables, it wasn’t as significant, I think, as it is now, like before it wasn’t as much money. So, I could probably get maybe a few bananas or whatever like that, but now I can get like a lot of fruit and a lot of vegetables, and it’s like really significant. $50 goes a long way for fresh fruit.”—Respondent 40
	“Now that like I said before, I do receive more, so I am capable of buying more.”—Respondent 20
More frequent shopping occasions	“Oh, he loves fruit. It allows me to get fruit twice throughout the month, rather than just the one time. It is a big help with dinner and getting and having broccoli and cabbage.”—Respondent 53
	“So, what I usually try to do is try to space it out. Like get as much as I know that they’re going to eat within a week or two and then go to the, go back to the grocery market and then like reup that’s how I usually do it. So, I feel like, you know, maybe $50 would be good for each person. because, like I said, you could, you could get a good amount for, you know, two weeks and then go back and spend another $25 on fruits and vegetables.”—Respondent 14
**Theme #2: Increased Consumption of Fruits and Vegetables**	
Healthier Diet	“Yeah, we eat healthier. It’s much healthier.”—Respondent 52
	“I guess we make healthier choices, since we have so much extra to spend on fruits and vegetables and I can make a lot more things from like scratch. I don’t have to buy as many frozen vegetables or canned vegetables.”—Respondent 9
**Theme #3: Enhanced Dietary Variety**	
More Variety	“Yeah, just give, you know, different variety. And like I said, I tried different fruit, you know, different things. So, it did help a lot with, you know, the little bit extra.”—Respondent 23
	“I do purchase different things. We try different vegetables, different fruits that my kids don’t usually eat on a normal day. Usually, we like with the low value, I get the kids’ favorites, cucumbers, strawberries, bell peppers, carrots. With the higher amount I’m able to change it around a little bit like tomatoes, zucchini, Brussel sprouts. My 11-year-old loves Brussel sprouts. It’s a vegetable. You can get it but It’s expensive. So, he barely, rarely gets those unless I have the extra money.”—Respondent 17
More Individualized	“If, when you go to a grocery market, $11 to 47, that makes a big difference that if each child in a household the choice and the option to go ahead and really have the options to eat and enjoy and get what they want. Like if my daughter says she wants strawberries, you know, she’s able to get that with hers. It’s just, my son said he want oranges and bananas. You know what I mean? Like, it, it gives the child, you know, in the household, the choice and option to go ahead and enjoy the, the fruits and vegetables.”—Respondent 14
	“It was great. Cause my five-year-old, she loved bananas, my son and my oldest, they loved apples and then the little halo. So, it was good. I was like, go ahead and pick out what you want.”—Respondent 28
**Theme #4: High Participant Valuation of the Increased CVB Allotment**	
CVB Participants’ Favorite Part of WIC	“So, yeah, I usually pretty much use my produce benefits to the, to the full extent of their abilities, because that’s my favorite part of WIC.”—Respondent 25
	“The fruits and vegetables I know that was fairly new from listening to people get WIC in the past because I was a new mom at this time and everybody, they didn’t always offer that. So, the addition of fruit and vegetables, probably the best thing WIC offers.”—Respondent 1
Saved Participants’ Own Money and/or their SNAP Benefits	“I use a lot of vegetables when I cook. So, it came in handy for me. Cause I didn’t have to use my food stamps. That was something that they would take care of. So, it helped me save on my food stamps for the meats and stuff. You know, other foods that we can’t get with WIC.”—Respondent 30
	“It [the CVB increase] kind of, it helps out a lot because I don’t have to spend that on like the fruit and vegetables. I won’t have to spend that on the [Electronic Benefits Transfer]. Like they kinda help out, I can get other stuff for the household.”—Respondent 14
Helped Participants	“They increased the amount of fruits and vegetables, I think before I was only getting like $11 and now it’s $24, so that’s, that helped a lot. I really like that.”—Respondent 52
	“The shifts in the fruit and vegetable benefit actually helped me out because my kids, they enjoy fruit. So, I was able to get more fresh fruits for them every week.”—Respondent 19

## Data Availability

The data presented in this study are available on request from the corresponding author.
